# Neuropathological Studies of Chickens Following Exposure to Chlorpyrifos

**DOI:** 10.4103/0971-6580.72675

**Published:** 2010

**Authors:** A. M. Kammon, R. S. Brar, S. Sodhi, H. S. Banga, H. S. Sandhu

**Affiliations:** Department of Veterinary Pathology, College of Veterinary Science, Guru Angad Dev Veterinary and Animal Sciences University, Ludhiana, India; 1 Department of Pharmacology and Toxicology, College of Veterinary Science, Guru Angad Dev Veterinary and Animal Sciences University, Ludhiana, India

**Keywords:** Chickens, chlorpyrifos, neuropathological effects

## Abstract

The objective of this study was to determine the putative neuropathological effects in young chickens after administration of a single dose of 55 mg/kg bw chlorpyrifos. The gross lesions of the nervous system comprised of congestions in the brain. Microscopic examination of brain showed mild congestion of cerebral blood vessels and mild perivascular cuffing of lymphomononuclear cells in the cerebral cortex and necrosis of the neurons. The interesting findings were the presence of cytoplasmic vacuolations of cerebral neurons and swelling of the endothelial cell of the cerebral capillaries. Cerebellum showed congestion and hemorrhages in the granular layer and necrosis of Purkinje cell. Sciatic nerve exhibited mild edema, swelling and degeneration of axons, and swelling of Schwann cells. There was a significant inhibition of plasma cholinesterase enzyme activity in chickens administered with chlorpyrifos compared to chickens of control group. The study revealed that administration of chlorpyrifos produces neuropathological lesions in chickens shortly after exposure.

## INTRODUCTION

Chlorpyrifos (CPF; diethyl 3, 5, 6-trichloro-2-pyridyl phosphorothionate; Dursban) is a broad-spectrum organophosphorus (OP) insecticide.[1] It is used extensively in agriculture throughout the world because of its high efficacy against pests. Acute or repeated low doses of some OP compounds have been associated with organophosphate-induced delayed neuropathy (OPIDN), characterized by distal degeneration of sensory and motor axons in peripheral nerves and spinal cord. This neurodegenerative disease is thought to be initiated by irreversible inhibition of a critical concentration of neurotoxic esterase (neuropathy target esterase) and by neuropathic OP compounds in neural tissue.[2] Previous experimental studies of OPIDN found wide differences in clinical signs and distribution of pathological lesions among animal species, strains, and ages, with the type and dosage.[3–6] There is contraindication regarding the effect of CPF to induce delayed neurotoxicity. CPF did not cause delayed neurotoxicity in White Leghorn adult hens after single doses up to 100 mg/kg.[7] In contrast, doses of CPF at about five times the oral LD_50_ in hens resulted in OPIDN, but at doses that required extensive and aggressive antidote treatment to keep the birds alive, both before treatment and throughout the treatment and recovery periods.[8]

The present study was undertaken to determine the neuropathological effects of a single dose of CPF in young chickens other than delayed neuropathy. The neuropathologic lesions in the central and peripheral nerves systems are documented.

## MATERIALS AND METHODS

A total of thirty 50-day-old layer chickens were randomly divided into two groups of 15 each. Chickens in group 1 were fasted for six hours before insecticide administration and received single dose of 55 mg/kg bw CPF orally. Group 2 served as a control. The chickens were closely watched for the presence of clinical signs. The experiment was performed after getting the permission from Institutional Animal Ethics Committee (IAEC), GADVASU, Ludhiana, India. The regulations addressing animal use and proper attention and care have been given to the chickens used in this experiment.

### Biochemical analysis

Blood samples were collected from all chickens of control group (n = 15) and from eight birds of CPF group immediately before death. Plasma was separated and immediately used for the analysis of acetylcholinesterase enzyme (AChE), using commercial kits (Bayer Diagnostics India Ltd, Baroda, Gujarat, India) on Biotron BTR-830 analyzer.

### Histopathological examination

Representative samples from brain and sciatic nerve were collected in 10% neutral formalin. After washing in running water followed by dehydration in alcohol, tissues were embedded and 5 *μ* paraffin sections were cut and stained with hematoxylin and eosin as per the method of Luna.[9]

## RESULTS AND DISCUSSION

CPF at the dose of 55 mg/kg orally produced cholinergic signs commencing two hours after administration which included excitation followed by sluggishness, watery diarrhea, excessive salivation changing to drooling, and rigid stance with dropping of wings. The chickens were unable to stand and showed paralysis and convulsions before death.

**Figure 1:**
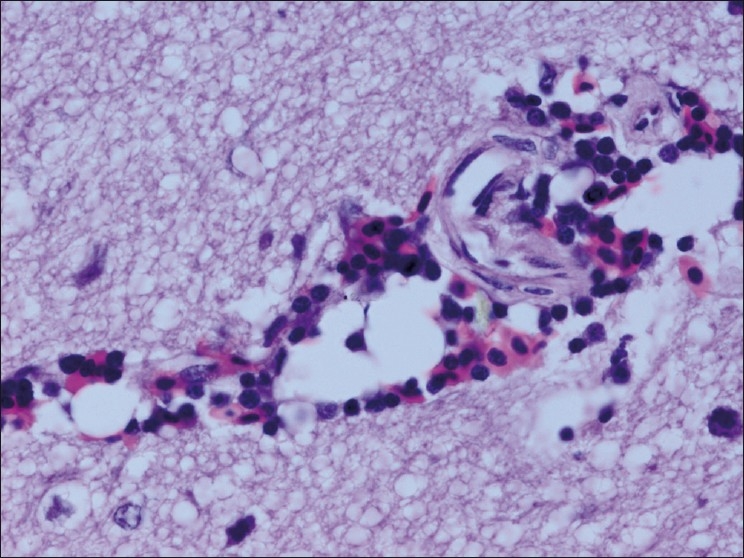
Brain showing mild perivascular cuffing of lymphomononuclear cells in the cerebral cortex H and E, ×100

**Figure 2:**
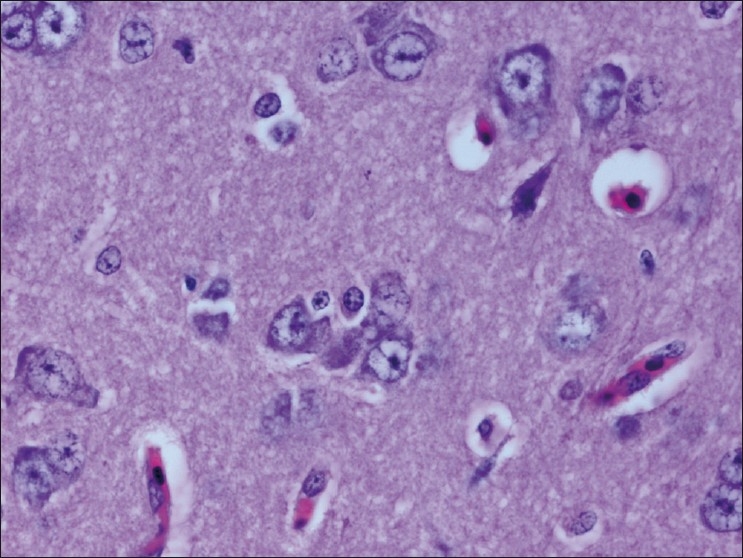
Cerebral neurons showing degeneration, cytoplasmic vacuolations, shrinkage of their size and necrosis H and E, ×100

**Figure 3:**
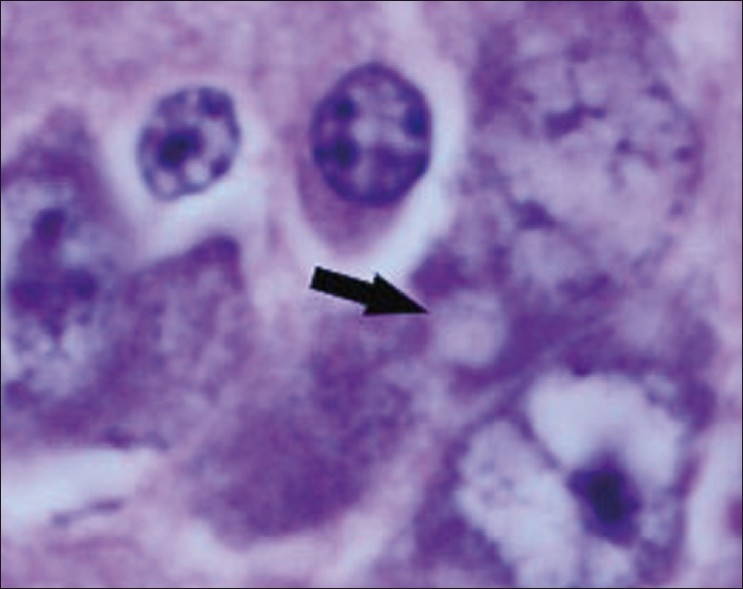
Higher magnification of Figure 2

**Figure 4:**
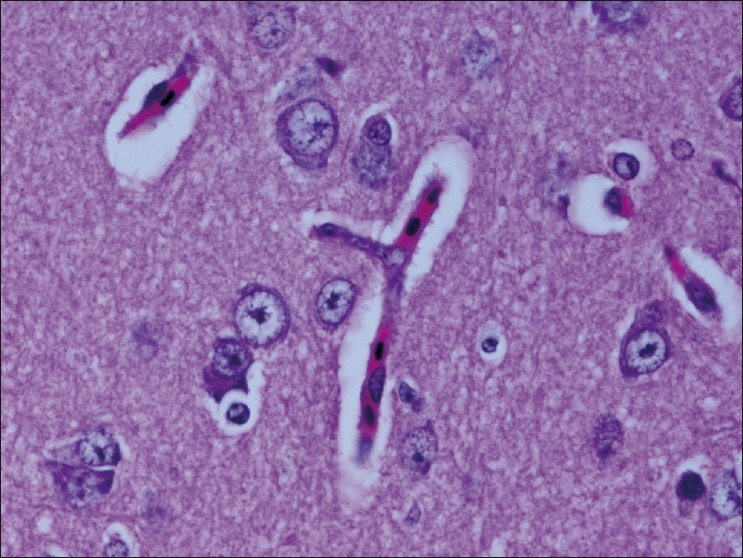
Cerebral endothelial cells became swollen and blocked the lumen of the capillary H and E, ×100

**Figure 5:**
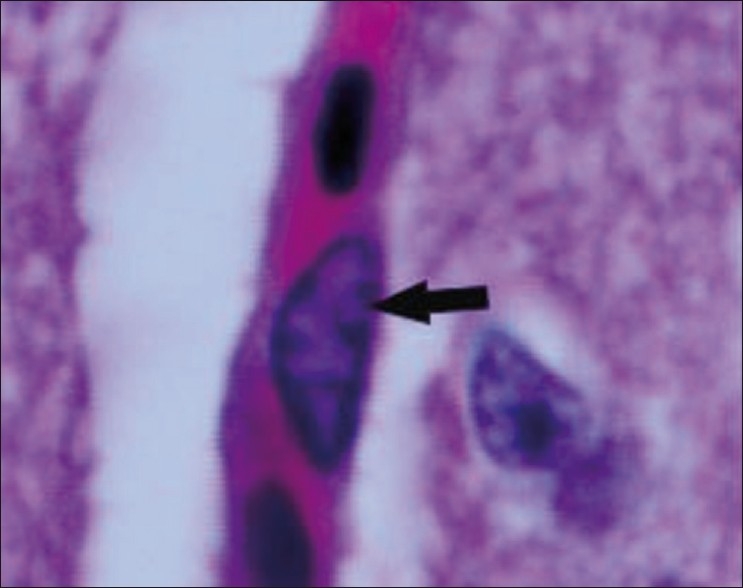
Higher magnification of Figure 4

**Figure 6:**
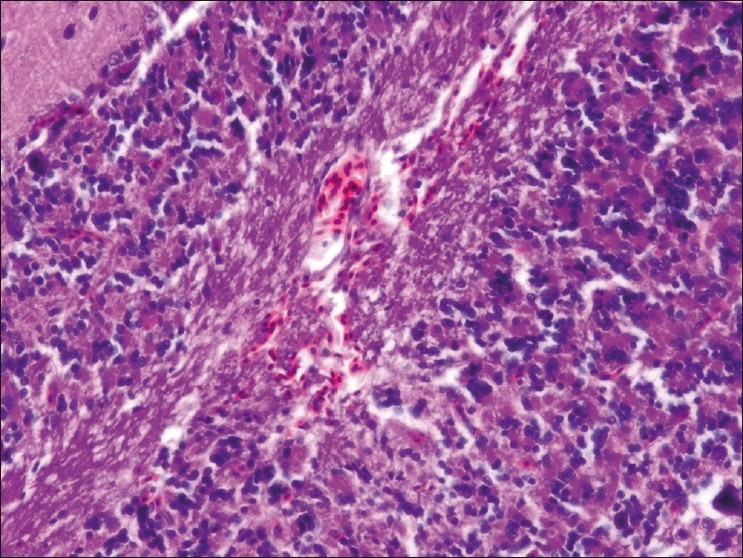
Congestion and hemorrhages in the granular layer of cerebellum H and E, ×40

**Figure 7:**
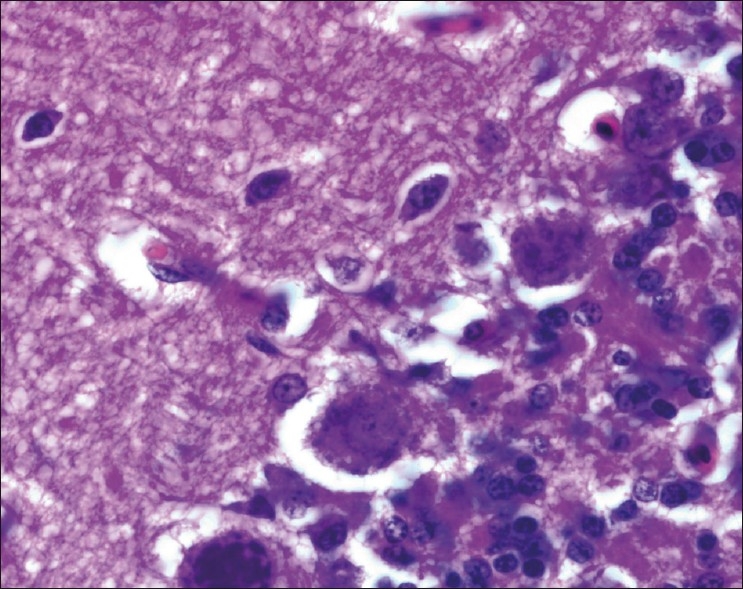
Degeneration of Purkinje cell H and E, ×100

**Figure 8:**
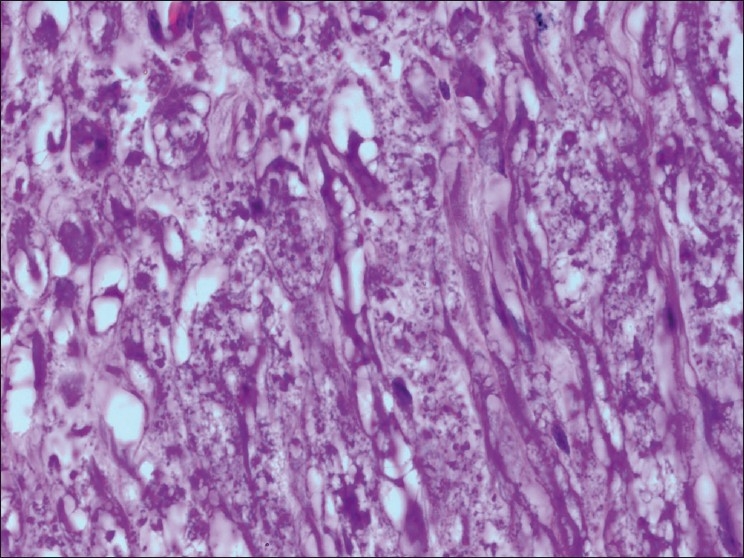
Section of sciatic nerve exhibiting mild edema, swelling and degeneration of axons and swelling of schwann cells H and S, ×100

The chickens were necropsied immediately after blood collection and after death. The sacrificed chicks were subjected to gross examination and tissues collected were used for histopathological findings. The gross lesions of the nervous system comprised of congestions in the brain. Microscopic examination of brain showed mild perivascular cuffing of lymphomononuclear cells in the cerebral cortex [Figure 1]. Interestingly, the cerebral neurons showed degeneration, formation of vacuoles in the cytoplasm, shrinkage of their size, and necrosis [Figure 2]. Higher magnification of these neurons is shown in Figure 3. However, similar vacuolization of the neuronal nuclei and cytoplasm was more pronounced, with predominance of large vacuoles in mice with serotonin deficiency. The revealed vacuolization in the neuronal nuclei and invagination of the nuclear membrane of these mice appeared to be due to impairment of synthetic processes.[10] In another study, topical application of methiocarb on the land snail *Eobania vermiculata* revealed highly vacuolated neuronal cytoplasm. Such vacuoles were regarded as a cellular defense mechanism against injurious substances that segregated in the vacuoles.[11] The second interesting finding in our study is that the cerebral endothelial cells became swollen and blocked the lumen of the capillary [Figure 4]. Higher magnification of cerebral endothelial cells is shown in Figure 5. However, Paljarvi *et al*.[12] reported ultrastructural changes in capillaries of rat cerebral cortex induced by ischemia and high lactic acidosis. Endothelial cells were swollen after five minutes of recirculation, and the changes grew markedly worse during 90 minutes of recirculation. Nuclear chromatin coarsened and mitochondria swelled up. Morphometry showed that the lumen narrowed as a result of endothelial swelling.[12] Similar changes were reported in GP8 rat brain endothelial cell cultures treated with mannitol.[13] Cerebellum showed congestion and hemorrhages in the granular layer [Figure 6] and degeneration of Purkinje cell [Figure 7]. It is clear that CPF at a dose of 55 mg/kg bw crosses the brain–blood barriers, causing direct damage to the capillaries and then to neurons. Sections of sciatic nerve exhibited mild edema, swelling and degeneration of axons, and swelling of Schwann cells [Figure 8].

In the present study, the neuropathological changes were associated with significant inhibition of plasma cholinesterase enzyme activity in chickens administered with CPF compared with chickens of control group. The insecticidal action of CPF stems from inhibition of AChE by CPF -oxon, resulting in acute cholinergic toxicity.[1]

Usually, single neurotoxic doses of organophosphates in chicken give rise to nerve fiber degeneration after a latent period of one week or more.[14] However in our study, neuropathological lesions were found shortly after administration of a single dose of 55 mg/kg bw CPF, which are in consistent with lesions of positive control white leghorn hens given tri-o-cresyl phosphate at a dose of 232 mg/kg bw, but birds treated with CPF at 50 or 100 mg/kg bw had a low incidence of focal lymphocytic infiltrate in the sciatic nerve without any other adverse neuropathological findings.[7] In contrast, the oral administration of a single dose of CPF (110 mg/kg bw) to hens, followed by a repeated dose after 21 days, did not produce any clinical signs of delayed neurotoxicity, and this result was confirmed by histopathological examination, which showed no treatment-related change in nerve tissues as a result of CPF administration.[15] In another study, doses of CPF at about five times the oral LD_50_ in hens resulted in OPIDN, but at doses that required extensive and aggressive antidote treatment to keep the birds alive, both before treatment and throughout the treatment and recovery periods.[8]

In conclusion, CPF produced neuropathological lesions in chickens shortly after administration. However, the neuropathogenesis of CPF toxicity in chickens needs to be elucidated.
